# Systemic pro-inflammatory response identifies patients with cancer with adverse outcomes from SARS-CoV-2 infection: the OnCovid Inflammatory Score

**DOI:** 10.1136/jitc-2020-002277

**Published:** 2021-03-22

**Authors:** Gino M Dettorre, Saoirse Dolly, Angela Loizidou, John Chester, Amanda Jackson, Uma Mukherjee, Alberto Zambelli, Juan Aguilar-Company, Mark Bower, Christopher C T Sng, Ramon Salazar, Alexia Bertuzzi, Joan Brunet, Ricard Mesia, Ailsa Sita-Lumsden, Elia Seguí, Federica Biello, Daniele Generali, Salvatore Grisanti, Pavetha Seeva, Gianpiero Rizzo, Michela Libertini, Antonio Maconi, Charlotte Moss, Beth Russell, Nadia Harbeck, Bruno Vincenzi, Rossella Bertulli, Diego Ottaviani, Raquel Liñan, Andrea Marrari, M Carmen Carmona-García, Neha Chopra, Carlo Alberto Tondini, Oriol Mirallas, Valeria Tovazzi, Vittoria Fotia, Claudia Andrea Cruz, Nadia Saoudi-Gonzalez, Eudald Felip, Ariadna Roqué, Alvin J X Lee, Tom Newsom-Davis, David García-Illescas, Roxana Reyes, Yien Ning Sophia Wong, Daniela Ferrante, Lorenza Scotti, Javier Marco-Hernández, Isabel Ruiz-Camps, Andrea Patriarca, Lorenza Rimassa, Lorenzo Chiudinelli, Michela Franchi, Armando Santoro, Aleix Prat, Alessandra Gennari, Mieke Van Hemelrijck, Josep Tabernero, Nikolaos Diamantis, David J Pinato, OnCovid study group

**Affiliations:** 1 Department of Surgery and Cancer, Imperial College London, Hammersmith Hospital, London, UK; 2 Medical Oncology, Guy’s and St Thomas’ NHS Foundation Trust (GSTT), London, UK; 3 Department of Infectious Diseases, Internal Medicine, Institut Jules Bordet, Université Libre de Bruxelles, Brussels, Belgium; 4 Medical Oncology, School of Medicine, Cardiff University, Cardiff, UK; 5 Medical Oncology, Velindre Cancer Centre, Cardiff, UK; 6 Clinical Trials, Velindre Cancer Centre, Cardiff, UK; 7 Medical Oncology, Barts Health NHS Trust, London, UK; 8 Oncology Unit, ASST Papa Giovanni XXIII, Bergamo, Italy; 9 Medical Oncology, Vall d'Hebron University Hospital and Institute of Oncology (VHIO), Barcelona, Spain; 10 Infectious Diseases, Vall d'Hebron University Hospital, Barcelona, Spain; 11 Department of Oncology and National Centre for HIV Malignancy, Chelsea and Westminster Hospital, London, UK; 12 Cancer Division, University College London Hospitals, London, UK; 13 Department of Medical Oncology, ICO L’Hospitalet, Oncobell Program (IDIBELL), CIBERONC, Hospitalet de Llobregat, Spain; 14 Medical Oncology and Hematology Unit, Humanitas Cancer Center, Humanitas Clinical and Research Center - IRCCS, Rozzano, Milan, Italy; 15 Department of Medical Oncology, Catalan Institute of Oncology, University Hospital Josep Trueta, Girona, Spain; 16 Department of Medical Oncology, Catalan Institute of Oncology, Badalona, Spain; 17 Department of Medical Oncology, Hospital Clinic, Barcelona, Spain; 18 Division of Oncology, Department of Translational Medicine, University of Piemonte Orientale and Maggiore della Carità Hospital, Novara, Italy; 19 Multidisciplinary Breast Pathology and Translational Research Unit, ASST Cremona, Cremona, Italy; 20 Department of Medical, Surgical and Health Sciences, University of Trieste, Trieste, Italy; 21 Medical Oncology Unit, Spedali Civili, Brescia, Italy; 22 Medical Oncology Unit, Fondazione IRCCS Policlinico San Matteo, Pavia, Italy; 23 Medical Oncology Unit, Fondazione Poliambulanza Istituto Ospedaliero, Brescia, Italy; 24 Infrastruttura Ricerca Formazione Innovazione, Azienda Ospedaliera SS Antonio e Biagio e Cesare Arrigo, Alessandria, Italy; 25 Translational Oncology and Urology Research (TOUR), School of Cancer and Pharmaceutical Sciences, King’s College London, London, UK; 26 Department of Gynecology and Obstetrics, Breast Center and Gynecological Cancer Center and CCC Munich, University Hospital Munich, Munich, Germany; 27 Medical Oncology, Policlinico Universitario Campus Bio-Medico, Rome, Italy; 28 Medical Oncology, Fondazione IRCCS Istituto Nazionale dei Tumori, Milano, Italy; 29 Department of Translational Medicine, Unit of Cancer Epidemiology, CPO-Piemonte, University of Eastern Piedmont, Novara, Italy; 30 Department of Internal Medicine, Hospital Clinic, Barcelona, Spain; 31 Division of Haematology, Department of Translational Medicine, University of Piemonte Orientale and Maggiore della Carità Hospital, Novara, Italy; 32 Department of Biomedical Sciences, Humanitas University, Via Rita Levi Montalcini, 20090 Pieve Emanuele, Milan, Italy; 33 Translational Genomics and Targeted Therapies in Solid Tumors, IDIBAPS, Barcelona, Spain; 34 Medical Oncology, Vall d'Hebron University Hospital and Institute of Oncology (VHIO), IOB-Quiron, UVic-UCC, Barcelona, Spain

**Keywords:** inflammation, inflammation mediators, COVID-19

## Abstract

**Background:**

Patients with cancer are particularly susceptible to SARS-CoV-2 infection. The systemic inflammatory response is a pathogenic mechanism shared by cancer progression and COVID-19. We investigated systemic inflammation as a driver of severity and mortality from COVID-19, evaluating the prognostic role of commonly used inflammatory indices in SARS-CoV-2-infected patients with cancer accrued to the OnCovid study.

**Methods:**

In a multicenter cohort of SARS-CoV-2-infected patients with cancer in Europe, we evaluated dynamic changes in neutrophil:lymphocyte ratio (NLR); platelet:lymphocyte ratio (PLR); Prognostic Nutritional Index (PNI), renamed the OnCovid Inflammatory Score (OIS); modified Glasgow Prognostic Score (mGPS); and Prognostic Index (PI) in relation to oncological and COVID-19 infection features, testing their prognostic potential in independent training (n=529) and validation (n=542) sets.

**Results:**

We evaluated 1071 eligible patients, of which 625 (58.3%) were men, and 420 were patients with malignancy in advanced stage (39.2%), most commonly genitourinary (n=216, 20.2%). 844 (78.8%) had ≥1 comorbidity and 754 (70.4%) had ≥1 COVID-19 complication. NLR, OIS, and mGPS worsened at COVID-19 diagnosis compared with pre-COVID-19 measurement (p<0.01), recovering in survivors to pre-COVID-19 levels. Patients in poorer risk categories for each index except the PLR exhibited higher mortality rates (p<0.001) and shorter median overall survival in the training and validation sets (p<0.01). Multivariable analyses revealed the OIS to be most independently predictive of survival (validation set HR 2.48, 95% CI 1.47 to 4.20, p=0.001; adjusted concordance index score 0.611).

**Conclusions:**

Systemic inflammation is a validated prognostic domain in SARS-CoV-2-infected patients with cancer and can be used as a bedside predictor of adverse outcome. Lymphocytopenia and hypoalbuminemia as computed by the OIS are independently predictive of severe COVID-19, supporting their use for risk stratification. Reversal of the COVID-19-induced proinflammatory state is a putative therapeutic strategy in patients with cancer.

## Introduction

SARS-CoV-2 is the novel *Betacoronavirus* first identified in Wuhan, China, that has resulted in over 104 million cases and more than two million deaths globally as of February 3, 2021.^1 2^ COVID-19, the resulting disease from SARS-CoV-2 infection, is characterized by a myriad of symptoms and systemic complications. Defining symptoms according to WHO guidelines include fever, cough, and dyspnea, alongside a number of non-specific constitutional symptoms.^3^ Complications are similarly varied and system-wide: complicated COVID-19 involves acute respiratory distress syndrome (ARDS), coagulopathy, septic shock, and acute kidney and cardiac injuries.^1^

Evolving clinical experience in the management of SARS-CoV-2 has confirmed that lethality from COVID-19 is related not only to the cytopathic effect of the virus but also to the host’s response to infection, which leads to end-organ damage and mortality through ignition of a sustained systemic inflammatory response.^4^ Patients with severe COVID-19 display heightened blood levels of interleukin (IL)-6, IL-2, IL-7, IL-10, and chemokines such as monocyte chemoattractant protein-1, implicating the innate immune response in COVID-19 progression.^4^ Assessment of inflammatory status at the time of COVID-19 diagnosis may lend itself as a bedside prognostic marker and novel immunological domain for therapeutic targets.

Systemic inflammation is in fact a unifying mechanism between cancer progression and COVID-19. The acute-phase response (APR) is a series of coordinated immune and metabolic changes that the host orchestrates in response to potentially harmful insults. The host’s innate immune response is central to the coordination of the APR, which is linked to both infectious and non-infectious *noxae*, including cancer.^5^ Activation of an APR is reflected in the acute derangement of hematological and biochemical parameters, including reactive leukocytosis, peripheral blood neutrophilia, lymphopenia, elevated C-reactive protein (CRP) levels, and hypoalbuminemia.^5 6^ A number of these laboratory parameters have been individually linked to worse prognosis from COVID-19.^4^ Inflammation-based indices, including the neutrophil:lymphocyte ratio (NLR),^7^ platelet:lymphocyte ratio (PLR),^8^ Prognostic Nutritional Index (PNI),^9 10^ modified Glasgow Prognostic Score (mGPS),^11^ and Prognostic Index (PI),^12^ have been derived from optimal integration of acute-phase reactants and have been consistently shown to predict for adverse prognosis independent of treatment, stage, and type of cancer. Evaluation of these indices in the context of SARS-CoV-2-infected patients with cancer may elucidate parameters of the APR that are linked to the progression of SARS-CoV-2 infection in its most lethal forms. We took special interest in the PI, which we renamed the OnCovid Inflammatory Score (OIS) in the context of COVID-19, as its calculation considers lymphopenia and hypoalbuminemia, two parameters individually shown to predict for COVID-19 prognosis.^4^

In a previous study, we had shown that a significant proportion of patients with cancer develop severe COVID-19 and that mortality is strongly related to host rather than oncological factors, including number of comorbidities and advancing age.^13 14^ However, the mechanisms underlying excess mortality observed in patients with cancer are not fully understood. In this study, we sought to determine whether the presence of a systemic inflammatory response at the time of COVID-19 diagnosis may help identify patients with severe SARS-CoV-2 infection and predict outcome in this population. To pursue this aim, we independently validated the OIS and a panel of inflammation-based indices in a large repository of patients with COVID-19 and cancer as part of the OnCovid study.

## Materials and methods

### Study design, patient demographics, and data collection

From the OnCovid repository, a retrospective registry of SARS-CoV-2-infected patients with cancer in Europe,^14^ we aimed to evaluate a panel of inflammation-based indices that are linked with the survival of patients with cancer for their prognostic role in the context of COVID-19 diagnosis.^7–9 11 12^ For the purpose of this study, the combination of hypoalbuminemia and lymphocytopenia (albumin concentration (g/L)+5×total lymphocyte count (10^9^/L)), previously qualified as a nutritional predictor of outcome (PNI), was redefined as the OIS in view of its qualification as an inflammatory predictor in the context of COVID-19 infection. Between February 27 and June 23, 2020, 1318 patients were consecutively referred from 23 academic centers in the UK (n=539), Spain (n=380), Italy (n=374), Belgium (n=19), and Germany (n=6, (online supplemental table 1)). Eligibility criteria for OnCovid included (1) SARS-CoV-2 infection confirmed by nasopharyngeal swab and subsequent reverse transcriptase PCR (RT-PCR)^15^ and (2) history of solid or hematological malignancy at any timepoint before or during COVID-19 disease course. Both patients with active malignancies and patients in cancer remission were included in the study. Patients were ≥18 years of age, and anonymized electronic medical record data were entered into the Research Electronic Data Capture (Vanderbilt University) tool hosted by the Medical Statistics Unit in Novara, Italy,^16 17^ which coordinated database access and curation.

10.1136/jitc-2020-002277.supp1 Supplementary data

### Inflammation-based indices

Eligible patients (n=1071) were stratified on the basis of a selected panel of five biomarkers of systemic inflammation (NLR, PLR, OIS, mGPS, and PI) at three timepoints: at the last oncological follow-up prior to SARS-CoV-2 infection (pre-COVID-19), at COVID-19 diagnosis, and, in surviving patients, at the first oncological follow-up after SARS-CoV-2 infection with a documented negative SARS-CoV-2 swab. Calculations for each inflammation-based index are shown in online supplemental table 2. Patients were stratified into good versus poor risk subgroups on the basis of predefined categories for the mGPS and PI, depending on whether they had an abnormal CRP and/or albumin (for mGPS) or white cell count (for PI) at the point of biomarker measurement.^11 12^ For inflammatory biomarkers characterized by a continuous distribution, good and poor risk groups were identified by the median value of the distribution.

10.1136/jitc-2020-002277.supp2 Supplementary data

### Definitions and study endpoints

Features of severe COVID-19 such as ARDS and septic shock were defined according to WHO guidelines.^3^ Furthermore, all comorbid conditions, complications, and key COVID-19 symptoms were determined from WHO guidelines.^3^ Resolution of SARS-CoV-2 infection was confirmed via negative RT-PCR result following nasopharyngeal swab for surviving patients. Active malignancy was defined by presence of oncological disease according to clinical criteria for the corresponding tumor type. Patients categorized as undergoing active cancer therapy had received anti-cancer therapy within 4 weeks of COVID-19 diagnosis, and patients with no treatment history for cancer were categorized as treatment naïve. To evaluate the prognostic value of inflammation-based indices, we randomly divided eligible patients into training and validation sets. Primary study outcome was mortality. Overall survival (OS) was determined from the date of positive SARS-CoV-2 RT-PCR to the date of death or final follow-up.

### Statistical analyses

Data following a normal distribution are presented as mean with SD, and data differing from normal distribution are presented as median with IQR. The Mann-Whitney U test and the Wilcoxon signed-rank test were used as appropriate to compare the medians of non-normally distributed continuous data. Categorical variables, shown as absolute counts or percentages, were compared using Pearson’s χ^2^ test. Kaplan-Meier estimates with a log-rank test were employed for univariable survival plot analysis. Univariable HRs were calculated through logistic Cox regression, and multivariable HRs were calculated through Cox regression with conditional backward elimination using a removal value of 0.10 and entry value of 0.05. Harrell’s concordance index (C-index) was used to evaluate the predictive ability of inflammation-based indices and the multivariable model. 150 bootstrap samples were used to calculate the 95% CI for each C-index, estimate the optimism due to potential overfitting, and provide optimism-adjusted C-index values with corresponding CIs. A two-tailed p value of §amp;lt;0.05 was considered the threshold for statistical significance. Dataset management and statistical analyses were performed using Statistical Product and Service Solutions V.24 for Apple Macintosh OSX (IBM, Armonk, New York, USA). Figures were created in Prism V.8 for Apple Macintosh OSX (GraphPad, San Diego, California, USA).

### Patient involvement

Patients were involved via retrospective analysis of anonymized patient chart data. Patients did not participate in the planning of this study, and this study involved no interventions.

## Results

### Inclusion criteria, training and validation set assortment, and demographics

**Figure 1:**
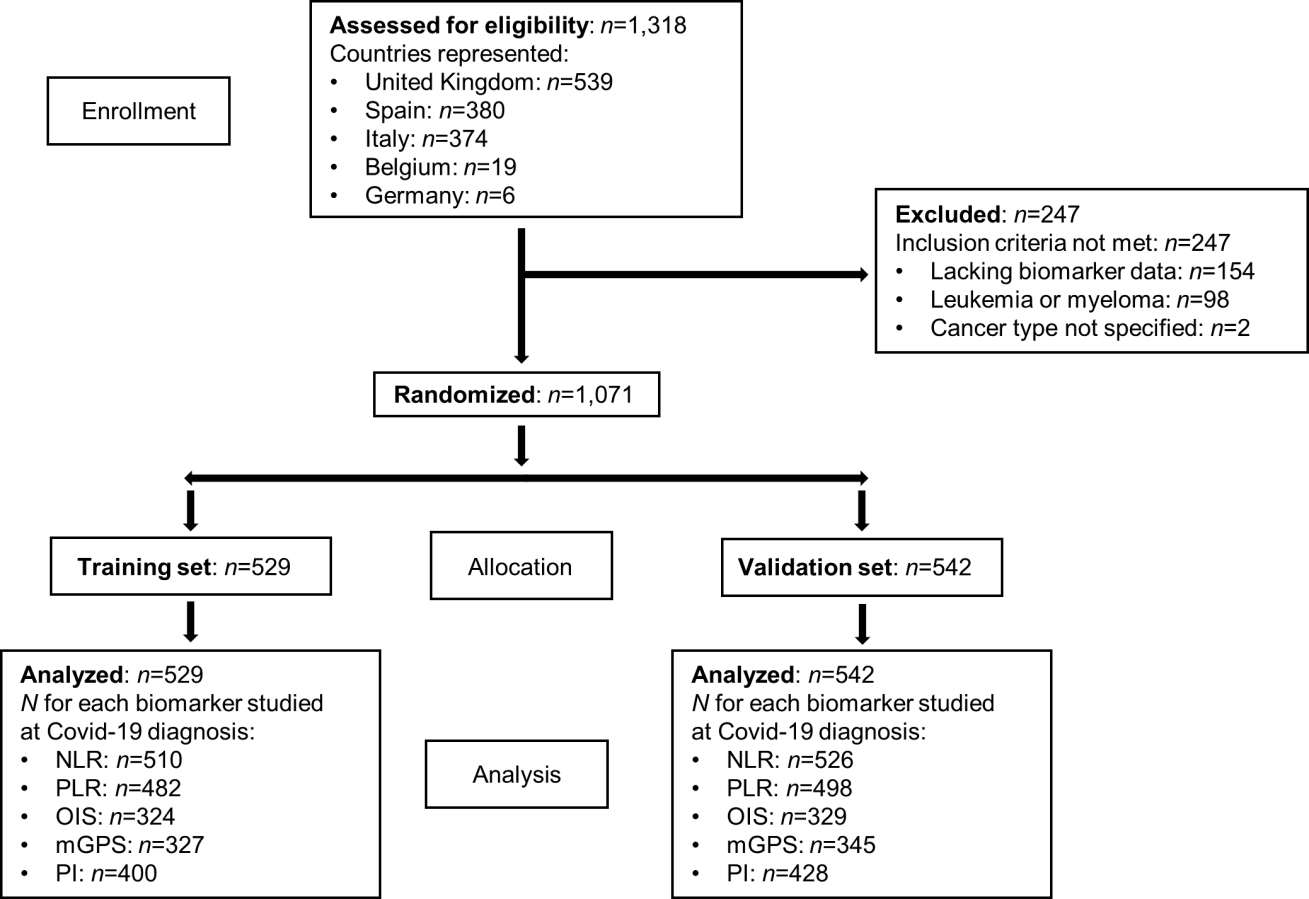
Patient disposition. mGPS, modified Glasgow Prognostic Score; NLR, neutrophil:lymphocyte ratio; OIS, OnCovid Inflammatory Score; PI, Prognostic Index; PLR, platelet:lymphocyte ratio.

A total of 1318 patients ≥18 years of age with a history of active malignancy or in cancer remission and possessing a positive COVID-19 diagnosis were consecutively entered into the main OnCovid registry. In total, 247 patients were excluded, leaving a final dataset of 1071 eligible patients (figure 1). Reasons for exclusion were (1) lack of laboratory data to calculate at least one biomarker of interest (n=154), (2) leukemia or myeloma diagnosis (n=98), and/or (3) lack of specification of cancer type (n=2). Patients with leukemia and myeloma were excluded due to the potential confounding effect of the neoplastic derangement on peripheral blood values used to compute bone marrow-derived inflammation indices.^18–20^ From this final study population (n=1071), patients were randomly allocated into a training set (n=529) or a validation set (n=542) matched for age, comorbid burden, number of COVID-19 complications, and proportion of patients with active malignancy (online supplemental table 3). Out of 1071 eligible patients, the majority were male (n=625, 58.3%) with a mean age of 68.2 years (SD ±13.4, range 21–99 years). Patient distribution across participating academic centers is shown in online supplemental table 1. Genitourinary cancers comprised the most common primary tumor site (n=216, 20.2%), and the majority of patients had evidence of active malignancy (n=687, 64.1%). At COVID-19 diagnosis, 519 (48.5%) patients had localized or locoregional disease; 420 patients (39.2%) had metastatic/advanced disease; and 516 (48.2%) patients were receiving anticancer therapy. Most patients had at least one comorbidity (n=844, 78.8%), most commonly hypertension (n=496, 46.3%) or cardiovascular disease (n=257, 24.0%).

10.1136/jitc-2020-002277.supp3 Supplementary data

Presenting SARS-CoV-2 symptoms included fever (n=660, 61.6%) and cough (n=542, 50.6%). Of the 957 (89.4%) patients hospitalized, 124 (13.0%) were escalated to intensive or subintensive care. The majority of patients required supplemental oxygen therapy (n=627, 58.5%), and a subset required mechanical ventilation (n=115, 10.7%). Most patients received at least one COVID-19-specific drug treatment (n=708, 66.1%), with the majority receiving an antimalarial or antiviral agent (n=622, 58.1%). A total of 754 (70.4%) patients developed COVID-19 complications, most frequently acute respiratory failure (n=627, 58.5%). At the time of database censoring (June 23, 2020), 375 (35.0%) patients were deceased and 680 (63.5%) were discharged from the hospital or were in-hospital survivors (online supplemental table 4 highlights COVID-19 complications and complete patient demographics).

10.1136/jitc-2020-002277.supp4 Supplementary data

### Systemic inflammatory response identifies patients with adverse outcomes

In eligible patients (n=1071), median NLR, PLR, and OIS at COVID-19 diagnosis were 6.1 (IQR 8.7), 270.1 (IQR 260.6), and 38.5 (IQR 10.5) respectively. In total, 526 (49.1%) patients had NLR≥6, 491 (45.8%) had PLR≥270, and 384 (35.9%) had OIS≤40. mGPS was available in 672 (62.7%) patients at COVID-19 diagnosis, with 503 (47.0%) patients being defined as intermediate (mGPS 1, n=196, 18.3%) or poor risk (mGPS 2, n=307, 28.7%). A total of 828 (77.3%) patients were evaluable for PI, with 686 (64.1%) classifying as intermediate (PI 1, n=546, 51.0%) or poor risk (PI 2, n=140, 13.1%) subgroups.

As shown in online supplemental figure 1, OIS values were significantly lower in patients with active malignancy (median 38.1, IQR 11.2), a factor known to predict for outcome from COVID-19,^13^ compared with patients in remission (median 39.5, IQR 9.6, p<0.05). Patients with active malignancy were also more likely to classify in poorer mGPS risk groups (p<0.05). A comparison of inflammation scores across tumor types is shown in online supplemental table 5.

10.1136/jitc-2020-002277.supp9 Supplementary data

10.1136/jitc-2020-002277.supp5 Supplementary data

**Figure 2:**
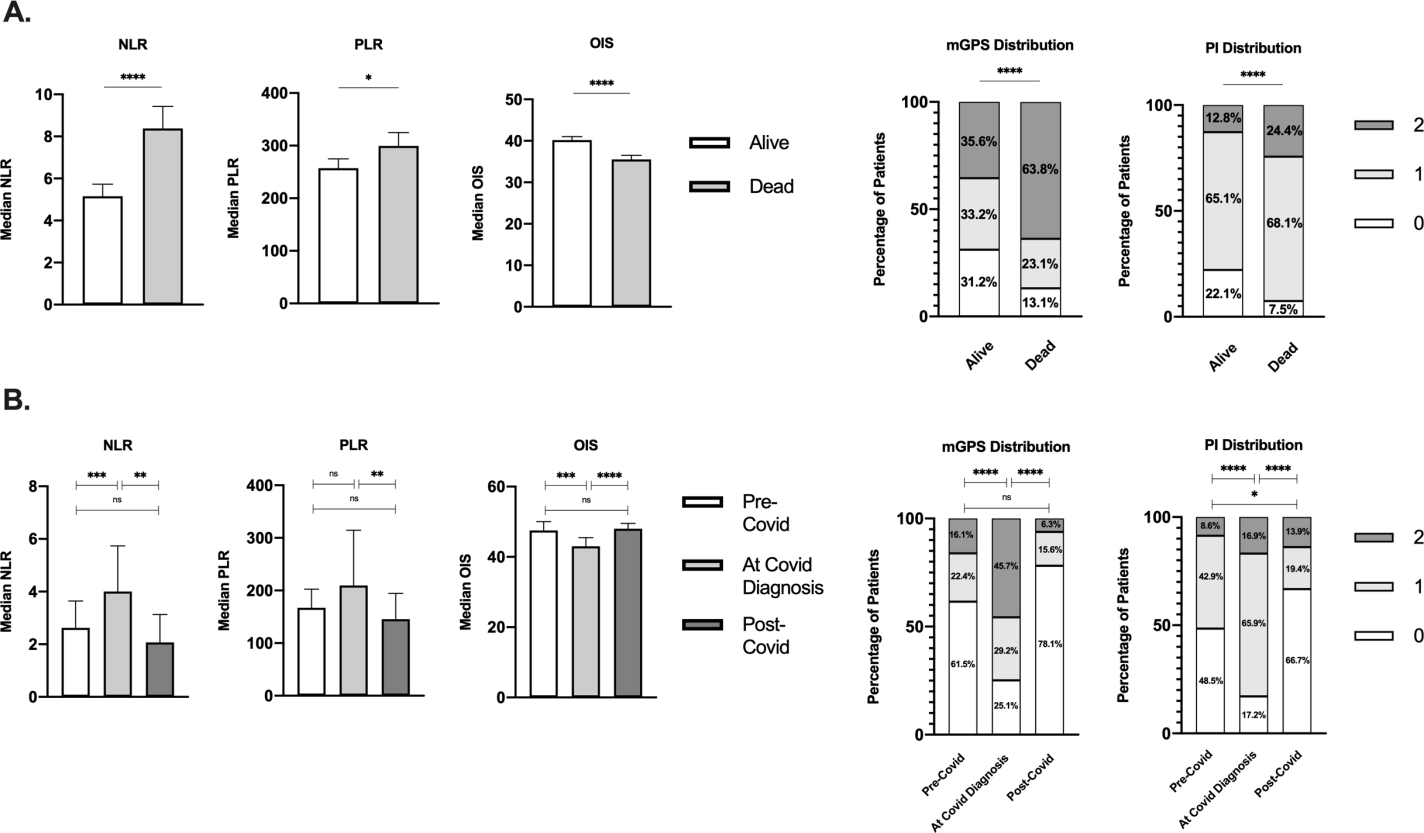
Relationship between inflammatory markers and COVID-19 outcomes and marker values at timepoints. (A) Median inflammatory index values or distributions at COVID-19 diagnosis in living versus deceased patients for the NLR (n=661 alive, n=360 dead; p§amp;lt;0.0001), PLR (n=628 alive, 338 dead; p§amp;lt;0.05), OIS (n=413 alive, n=230 dead; p§amp;lt;0.0001), mGPS (n=430 alive, n=229 dead; p§amp;lt;0.0001), and PI (n=525 alive, n=295 dead; p§amp;lt;0.0001). (B) Median values and distributions across timepoints for the NLR (n=55), PLR (n=50), OIS (n=23), mGPS (n=143 pre-COVID-19, n=672 at diagnosis, n=32 post-COVID-19), and PI (n=163 pre-COVID-19, n=828 at diagnosis, n=36 post-COVID-19). *P§amp;lt;0.05, **P§amp;lt;0.01, ***P§amp;lt;0.001, ****P§amp;lt;0.0001. Error bars represent 95% CIs from the median. mGPS, modified Glasgow Prognostic Score; NLR, neutrophil:lymphocyte ratio; ns, not significant; OIS, OnCovid Inflammatory Score; PI, Prognostic Index; PLR, platelet:lymphocyte ratio.

Median NLR, PLR, and OIS at COVID-19 diagnosis were studied in relation to patients’ mortality along with the distribution of the mGPS and PI. Mortality from COVID-19 was associated with categorization into the poorer risk group for each index (p§amp;lt;0.05, figure 2A).

To evaluate the relative contribution of active SARS-CoV-2 infection over underlying malignancy in determining the systemic inflammatory response, we evaluated dynamic changes across the pre-COVID-19, at, and post-COVID-19 diagnosis timepoints in a subset of SARS-CoV-2 survivors (n=65, 6.1%). All samples taken at post-COVID-19 diagnosis were defined as measurements taken during the first outpatient oncological follow-up after COVID-19 with a negative SARS-CoV-2 swab at the moment of blood sampling. Patients had data for all indices (NLR, n=55; PLR, n=50; OIS, n=23; mGPS, n=8; and PI, n=13). The mean interval between pre-COVID-19 and at COVID-19 diagnosis timepoints was 270 days (SD±855), and the mean interval between COVID-19 diagnosis and post-COVID-19 diagnosis timepoints was 39 days (SD±24).

As shown in figure 2B, NLR values were significantly higher at COVID-19 diagnosis (median 6.1, IQR 8.7) compared with pre-COVID-19 (median 2.9, IQR 3.1, p§amp;lt;0.001) and post-COVID-19 timepoints (median 2.6, IQR 2.8, p§amp;lt;0.01), whereas pre-COVID and post-COVID-19 values did not differ (p=0.82). Similar dynamic changes were described for the OIS, which appeared lower at COVID-19 diagnosis (median 38.5, IQR 10.5) compared with pre-COVID-19 (median 47.0, IQR 9.6, p§amp;lt;0.001) and post-COVID-19 timepoints (47.8, IQR 10.0, p§amp;lt;0.0001), with no difference noted between pre-COVID-19 and post-COVID-19 measurements (p=0.58). mGPS and PI distribution also varied across timepoints (figure 2B).

Next, we sought to determine the relationship between patients’ inflammatory scores at COVID-19 diagnosis and previously established predictors of mortality or disease severity.^13 21–24^ As shown in online supplemental table 6, patients in the poor risk groups for all inflammatory indices developed a greater number of COVID-19 complications. Furthermore, patients in poor risk groups for all indices except the PLR were older and had a higher comorbid burden. Patients in poor risk categories for the NLR, mGPS, and PI were less likely to be undergoing anticancer therapy at COVID-19 diagnosis and were more often male (online supplemental table 6).

10.1136/jitc-2020-002277.supp6 Supplementary data

We also investigated the association between inflammatory markers and laboratory variables known to predict for increased mortality from COVID-19.^21–24^ As shown in online supplemental figure 2, an NLR of ≥6 was associated with increased lactate dehydrogenase (LDH, p§amp;lt;0.0001), D-dimer (p§amp;lt;0.01), troponin (p<0.001), and ferritin (p<0.01) levels compared with an NLR<6. An OIS≤40 was associated with heightened LDH (p§amp;lt;0.001), D-dimer (p§amp;lt;0.001), and ferritin (p§amp;lt;0.001) levels. Poor risk mGPS and PI categories also exhibited an association with clinical variables.

10.1136/jitc-2020-002277.supp10 Supplementary data

### OIS predicts for decreased OS and greater risk of severe COVID-19 in patients with cancer

To accurately evaluate the prognostic performance of inflammation-based indices, we randomly assigned the 1071 eligible patients into a training (n=529) and validation set (n=542, figure 1). Both datasets were matched for key characteristics that are known to influence prognosis from COVID-19, including age ≥65 years, number of comorbidities, number of COVID-19 complications, and presence of active malignancy (online supplemental table 3).^13^ Table 1 presents complete demographics of both the training and validation sets. Unadjusted mortality rates were similar across datasets (36.1% in the training set and 35.0% in the validation set, p=0.16).

**Table 1:** Patient demographics of training and validation sets: features of patients from both the training (n=529) and validation (n=542) sets

Characteristics	Training set (total, n=529)	Validation set (total, n=542)
Age (years), mean±SD	67.9±13.3	68.5±13.5
Age 65 years, n (%)	311 (58.8)	334 (61.6)
Sex, n (%)		
Male	296 (56.0)	329 (60.7)
Female	231 (43.7)	212 (39.1)
Information unavailable	2 (0.3)	1 (0.2)
Smoking history, n (%)		
Never smoker	205 (38.8)	207 (38.2)
Current/former smoker	228 (43.1)	234 (43.2)
Unknown	96 (18.1)	101 (18.6)
Cancer type, n (%)		
Head and neck	22 (4.2)	12 (2.2)
Lung and thoracic	67 (12.7)	87 (16.1)
Gastroesophageal	30 (5.7)	22 (4.1)
Hepatobiliary	33 (6.2)	22 (4.1)
Duodenal and lower GI tract	61 (11.5)	71 (13.1)
Breast	85 (16.1)	92 (17.0)
Gynecological	35 (6.6)	22 (4.1)
Genitourinary	108 (20.4)	108 (19.9)
Skin	17 (3.2)	26 (4.8)
Lymphoma	49 (9.3)	38 (7.0)
Other	22 (4.1)	42 (7.6)
Tumor stage, n (%)		
Localized	173 (32.7)	187 (34.5)
Locoregional	72 (13.6)	87 (16.1)
Metastatic	223 (42.2)	197 (36.3)
Information unavailable	61 (11.5)	71 (13.1)
Tumor status at COVID-19 diagnosis, n (%)		
Active malignancy	353 (66.7)	334 (61.6)
Remission	150 (28.4)	182 (33.6)
Information unavailable	26 (4.9)	26 (4.8)
Ongoing anticancer therapy at COVID-19 diagnosis, n (%)	264 (49.9)	252 (46.5)
Prior radical therapies, n (%)	285 (53.9)	306 (56.5)
Surgery	244 (46.1)	266 (49.0)
Adjuvant/neoadjuvant chemotherapy	171 (32.3)	148 (27.3)
Prior palliative systemic therapy, n (%)	147 (27.8)	130 (24.0)
Chemotherapy	97 (18.3)	87 (16.1)
Immunotherapy	21 (4.0)	25 (4.6)
Endocrine therapy	29 (5.5)	24 (4.4)
Targeted therapy	22 (4.2)	23 (4.2)
Prior curative systemic therapy, n (%)	32 (6.0)	30 (5.5)
Prior radiotherapy, n (%)	150 (28.4)	169 (31.2)
Prior lines of palliative therapy, n (%)		
1	75 (14.2)	60 (11.1)
2	30 (5.7)	29 (5.4)
3	33 (6.2)	28 (5.2)
Comorbidities, n (%)	425 (80.3)	419 (77.3)
Hypertension	251 (47.4)	245 (45.2)
Diabetes	115 (21.7)	123 (22.7)
Cardiovascular disease	128 (24.2)	129 (23.8)
Chronic pulmonary disease	80 (15.1)	80 (14.8)
Chronic kidney disease	62 (11.7)	63 (11.6)
Cerebrovascular disease	37 (7.0)	41 (7.6)
Dementia	27 (5.1)	36 (6.6)
Peripheral vascular disease	19 (3.6)	21 (3.9)
Liver impairment	11 (2.1)	10 (1.8)
Immunosuppression	16 (3.0)	29 (5.4)
Steroid therapy in progress	23 (4.3)	27 (5.0)
Other	164 (31.0)	144 (26.6)
Number of comorbidities (%)		
0	104 (19.7)	123 (22.7)
1	149 (28.2)	149 (27.5)
2	129 (24.4)	118 (21.8)
3	147 (27.7)	152 (28.0)
COVID-19 symptoms at diagnosis, n (%)	497 (94.0)	514 (94.8)
Fever	317 (59.9)	343 (63.3)
Cough	257 (48.6)	285 (52.6)
Dyspnea	206 (38.9)	232 (42.8)
Fatigue	132 (25.0)	125 (23.1)
Myalgia	60 (11.3)	57 (10.5)
Diarrhea	50 (9.5)	76 (14.0)
Coryzal symptoms	23 (4.3)	26 (4.8)
Nausea or vomiting	33 (6.2)	47 (8.7)
Sore throat	18 (3.4)	9 (1.7)
Headache	21 (4.0)	21 (3.9)
Dysgeusia	14 (2.6)	21 (3.9)
Anosmia	13 (2.5)	16 (3.0)
Other (ie, confusion and delirium)	119 (22.5)	124 (22.9)
Number of symptoms at diagnosis (%)		
0	32 (6.0)	28 (5.2)
1	125 (23.6)	104 (19.2)
2	151 (28.5)	149 (27.5)
3	221 (41.9)	261 (48.2)
Hospitalization rate, n (%)	477 (90.2)	480 (88.6)
Admission to intensive or subintensive care unit, n (%)	59/477 (12.4)	65/480 (13.5)
COVID-19-specific drug treatments, n (%)	349 (66.0)	359 (66.2)
Antibiotics	294 (55.6)	301 (55.5)
Hydroxychloroquine or chloroquine	199 (37.6)	193 (35.6)
Lopinavir/ritonavir	86 (16.3)	86 (15.9)
Systemic corticosteroids	45 (8.5)	46 (8.5)
Remdesivir	5 (0.9)	7 (1.3)
Tocilizumab	18 (3.4)	28 (5.2)
Others	57 (10.8)	71 (13.1)
COVID-19-specific oxygen interventions, n (%)	309 (58.4)	323 (59.6)
Oxygen therapy	307 (58.0)	320 (59.0)
Mechanical ventilation	44 (8.3)	71 (13.1)
COVID-19 complications, n (%)	372 (70.3)	382 (70.5)
Acute respiratory failure	307 (58.0)	320 (59.0)
ARDS	61 (11.5)	70 (12.9)
Acute kidney injury	42 (7.9)	44 (8.1)
Secondary infection	49 (9.3)	37 (6.8)
Sepsis	29 (5.5)	23 (4.2)
Septic shock	22 (4.2)	23 (4.2)
Acute cardiac injury	12 (2.3)	12 (2.2)
Acute liver injury	4 (0.8)	6 (1.1)
Others (ie, DIC)	33 (6.2)	28 (5.2)
Number of complications (%)		
0	157 (29.7)	160 (29.5)
1	190 (35.9)	204 (37.6)
2	101 (19.1)	93 (17.2)
3	42 (7.9)	47 (8.7)
Information unavailable	39 (7.4)	38 (7.0)

ARDS, acute respiratory distress syndrome; DIC, disseminated intravascular coagulation; GI, gastrointestinal.

**Figure 3:**
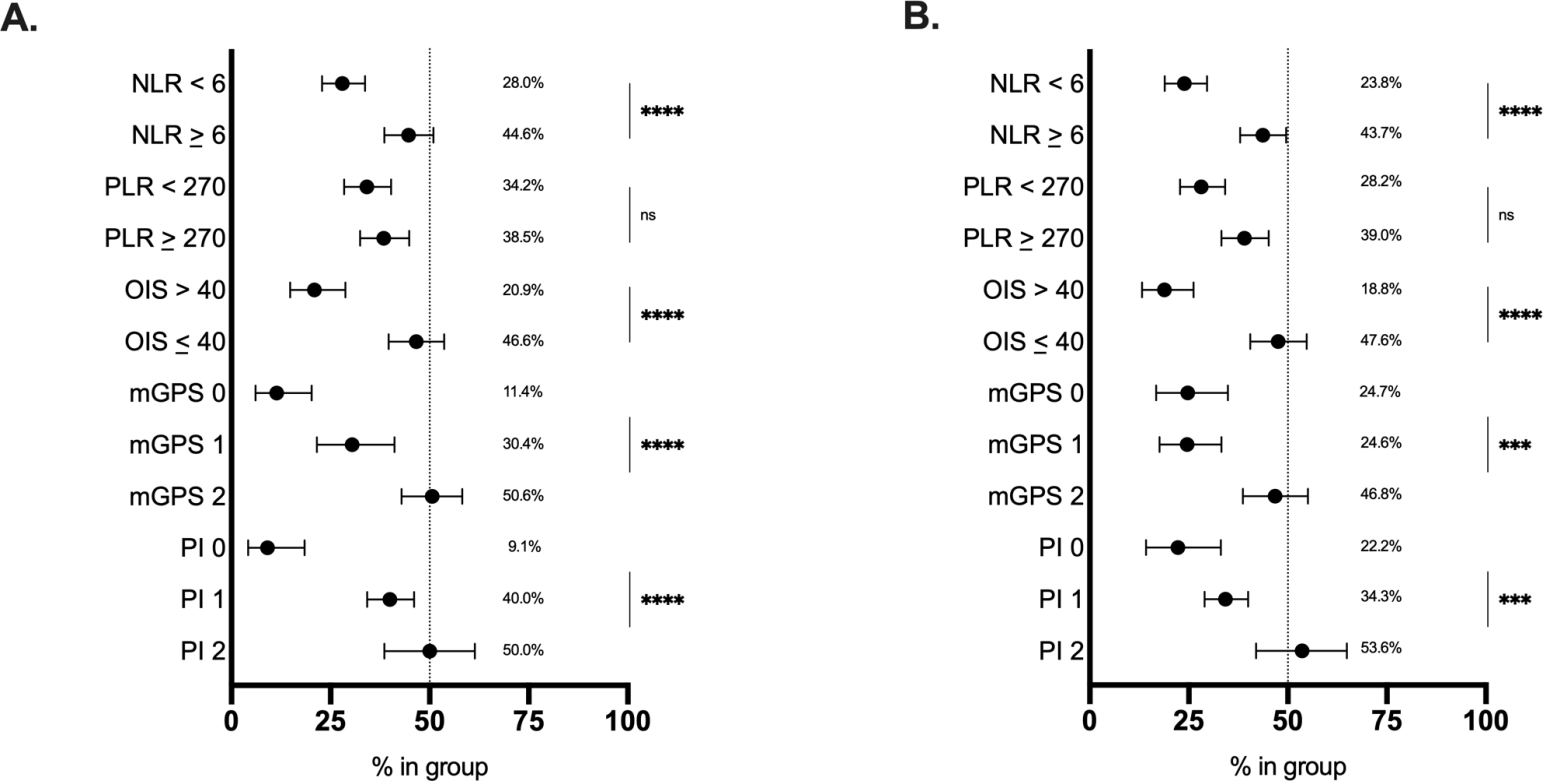
Relationship between inflammatory markers and unadjusted mortality. Inflammatory marker values at COVID-19 diagnosis divided into good and poor risk groups plotted against unadjusted mortality rates for the (A) training set and (B) validation set. Error bars represent lower and upper limits. ***P<0.001, ****P<0.0001. mGPS, modified Glasgow Prognostic Score; NLR, neutrophil:lymphocyte ratio; ns, not significant; OIS, OnCovid Inflammatory Score; PI, Prognostic Index; PLR, platelet:lymphocyte ratio.

**Figure 4:**
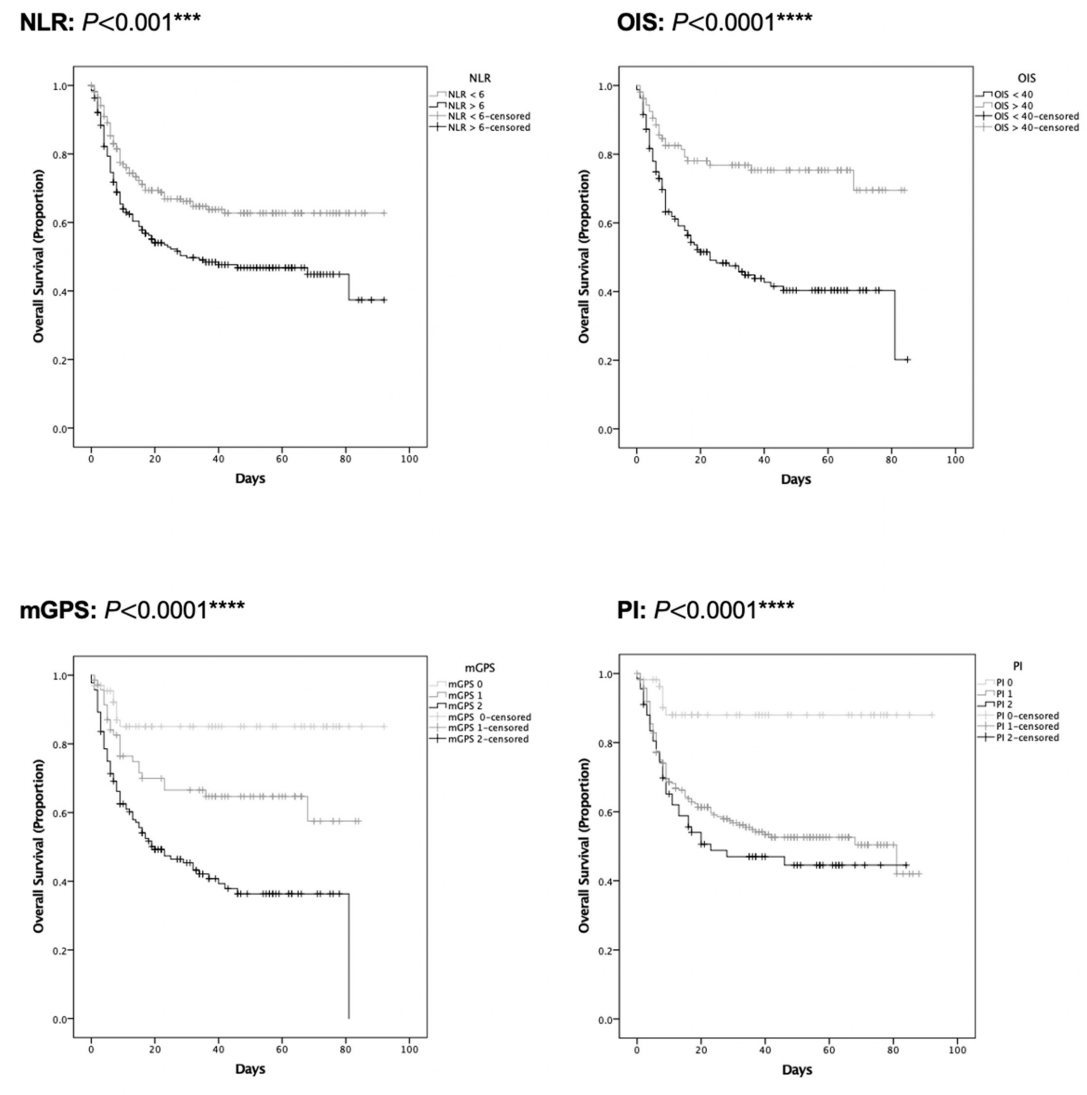
Univariable survival analysis of inflammatory markers (training set). Kaplan-Meier estimates for univariable survival analysis are shown for the NLR (n=447, p§amp;lt;0.001), OIS (n=273, p§amp;lt;0.0001), mGPS (n=278, p§amp;lt;0.0001), and PI (n=361, p§amp;lt;0.0001) at COVID-19 diagnosis with significance calculated following log-rank methodology. ***P§amp;lt;0.001, ****P§amp;lt;0.0001. mGPS, modified Glasgow Prognostic Score; NLR, neutrophil:lymphocyte ratio; OIS, OnCovid Inflammatory Score; PI, Prognostic Index.

**Figure 5:**
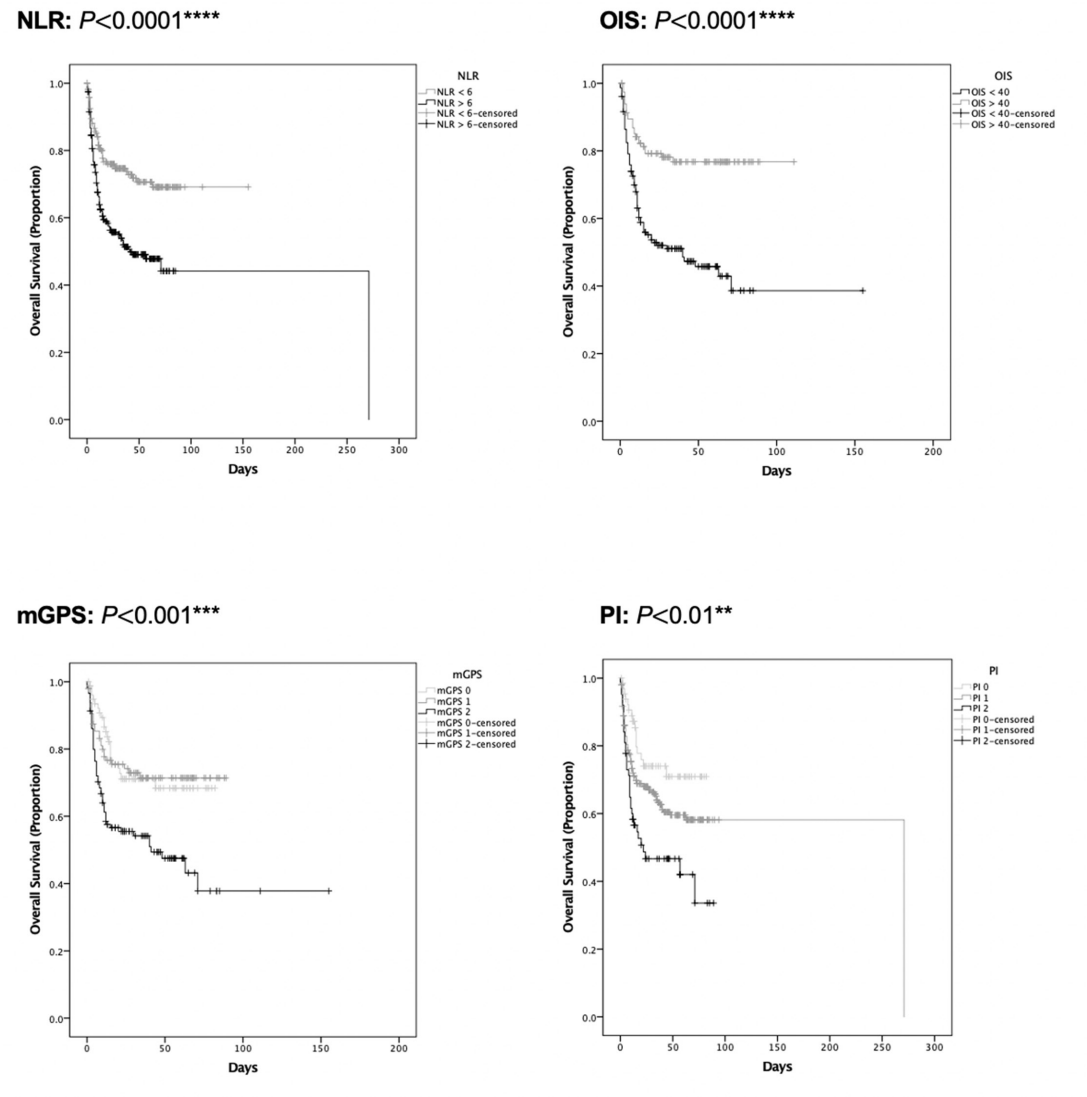
Univariable survival analysis of inflammatory markers (validation set). Kaplan-Meier estimates for univariable survival analysis are shown for the NLR (n=452, p§amp;lt;0.0001), OIS (n=272, p§amp;lt;0.0001), mGPS (n=291, p§amp;lt;0.001), and PI (n=384, p§amp;lt;0.01) at COVID-19 diagnosis with significance calculated following log-rank methodology. **P§amp;lt;0.01, ***P§amp;lt;0.001, ****P§amp;lt;0.0001. mGPS, modified Glasgow Prognostic Score; NLR, neutrophil:lymphocyte ratio; OIS, OnCovid Inflammatory Score; PI, Prognostic Index.

In the training set, all tested inflammatory indices but the PLR were associated with unadjusted mortality rates (p<0.0001), as shown in figure 3A. Analysis of unadjusted mortality in the validation set confirmed these findings (figure 3B). Patients with NLR of ≥6, OIS of ≤40, and poor risk mGPS and PI values had shorter median OS (p§amp;lt;0.001, figure 4 and online supplemental table 7). Additionally, as shown in table 2, patients in poor risk groups for all inflammatory indices except the PLR (p=0.55) presented univariable HRs above 1.00 (p≤0.001). This was confirmed in the validation set analysis (p<0.01; figure 5, table 2 and online supplemental table 7).

10.1136/jitc-2020-002277.supp7 Supplementary data

**Table 2:** Univariable and multivariable HRs for prognostic factors

	Training	n=529			Validation	n*=*542		
Prognostic factor	Univariable HR (95% CI)	P value	Multivariable HR (95% CI)	P value	Univariable HR (95% CI)	P value	Multivariable HR (95% CI)	P value
NLR <6/≥6	***1.68 (1.25 to 2.27)***	***0.001***	0.97 (0.60 to 1.55)	0.882	***2.07 (1.50 to 2.86)***	***<0.0001***	0.75 (0.45 to 1.26)	0.275
PLR <270/≥270	1.10 (0.81 to 1.45)	0.550	0.76 (0.49 to 1.19)	0.234	***1.55 (1.12 to 2.13)***	***0.008***	0.94 (0.52 to 1.70)	0.842
OIS >40/≤40	***2.78 (1.78 to 4.34)***	***<0.0001***	***1.97 (1.19 to 3.26)***	***0.008***	***2.83 (1.80 to 4.45)***	***<0.0001***	***2.48 (1.47 to 4.20)***	***0.001***
mGPS 0/2	***5.12 (2.57 to 10.22)***	***<0.0001***	2.35 (0.77 to 7.14)	0.132	***2.18 (1.32 to 3.60)***	***0.002***	1.37 (0.18 to 10.78)	0.764
PI 0/2	***5.61 (2.36 to 13.34)***	***<0.0001***	1.41 (0.13 to 15.76)	0.781	***2.71 (1.49 to 4.90)***	***0.001***	1.76 (0.74 to 4.17)	0.200
Sex (Female/male)	***1.38 (1.03 to 1.862)***	***0.032***	0.69 (0.45 to 1.07)	0.097	***1.40 (1.02 to 1.92)***	***0.039***	0.86 (0.51 to 1.45)	0.576
Age <65/≥65	***2.22 (1.60 to 3.06)***	***<0.0001***	1.47 (0.93 to 2.33)	0.103	***2.56 (1.78 to 3.67)***	***<0.0001***	***2.80 (1.50 to 5.22)***	***0.001***
Comorbid burden 0/≥2	***1.86 (1.24 to 2.80)***	***0.003***	1.28 (0.71 to 2.30)	0.412	***2.40 (1.53 to 3.76)***	***<0.0001***	2.19 (0.89 to 5.39)	0.090
Malignancy status Remission/active	***1.55 (1.09 to 2.21)***	***0.015***	1.60 (0.95 to 2.72)	0.080	1.20 (0.86 to 1.65)	0.284	1.16 (0.69 to 1.95)	0.575
Anticancer therapy at COVID-19 diagnosis No/yes	***0.59 (0.44 to 0.79)***	***<0.001***	0.65 (0.41 to 1.02)	0.063	***0.72 (0.53 to 0.97)***	***0.032***	0.94 (0.54 to 1.63)	0.813

Prognostic factors for both the training (n=529) and validation (n=542) are shown with their corresponding univariable and multivariable HRs with 95% CIs and p values. Univariable HRs were calculated through logistic Cox regression, and multivariable HRs were calculated through Cox regression with conditional backward elimination. Statistical signifiance indicated via bold and italics. HR, hazard ratio; NLR, neutrophil:lymphocyte ratio; PLR, platelet:lymphocyte ratio; OIS, OnCovid Inflammatory Score; mGPS, modified Glasgow Prognostic Score; PI, Prognostic Index.

Bold and italicized values are statistically significant (P<0.05).

mGPS, modified Glasgow prognostic score; NLR, neutrophil:lymphocyte ratio; OIS, OnCovid inflammatory score; PI, prognostic index; PLR, platelet:lymphocyte ratio.

In multivariable Cox regression models, the inflammatory indices were evaluated for their independent prognostic ability against established prognostic factors for COVID-19.^13 21–24^ In the training set analysis, the OIS was the only factor emerging as significantly prognostic with patients in the poor risk group (OIS≤40) presenting a multivariable HR of 1.97 (95% CI 1.19 to 3.26, p=0.008; table 2). The independent prognostic value of the OIS also emerged in multivariable analyses of the validation set (OIS≤40, HR 2.48, 95% CI 1.47 to 4.20, p=0.001; table 2), confirming its external validity as a prognostic factor for COVID-19. Analysis of inflammatory indices for prognostic ability was further evaluated through calculation of Harrell’s C-index (online supplemental table 8). The mGPS (optimism-adjusted C-index 0.634, 95% CI 0.595 to 0.680) and OIS (adjusted C-index 0.603, 95% CI 0.555 to 0.646) emerged as most predictive in the training set. OIS was most predictive in validation set analysis (adjusted C-index 0.611, 95% CI 0.564 to 0.668) followed by the mGPS (adjusted C-index 0.596, 95% CI 0.548 to 0.651; online supplemental table 8).

10.1136/jitc-2020-002277.supp8 Supplementary data

## Discussion

Previous work has emphasized the contribution of comorbid burden, advanced age, and a diagnosis of hematological malignancy as risk factors for mortality from COVID-19; however, the molecular and immunological mechanisms that lead to a more adverse COVID-19 course in patients with cancer are largely unknown.^13 25^

In our study, we demonstrate that the presence of a proinflammatory diathesis as measured by the NLR, OIS, mGPS and PI is generally associated with worsening levels of established biomarkers of COVID-19 severity such as higher circulating D-dimer, troponin, ferritin and LDH concentration and less strongly related to the presence of measurable or active malignancy. These findings, paired with evidence of dynamic changes of inflammatory markers, strengthen the link between systemic inflammation as a SARS-CoV-2-driven disease mechanism linked with adverse clinical outcome.

Interestingly, activation of a systemic inflammatory response appears unevenly distributed across tumor sites. Patients with breast cancer, for instance, had lower mortality in our study (20.9%) and were more likely to cluster within better risk categories according to inflammatory prognostic indices. The relationship between inflammatory scores and mortality in selected tumors such as breast cancer is highly provocative and warrants further research into whether certain tumor types are associated with a more favorable immune response to COVID-19.

Irrespective of tumor-specific considerations, analysis of survival confirms the NLR, OIS, mGPS, and PI as tumor-agnostic predictors of OS in both the training and validation sets, strengthening the external validity of the APR as a driver of mortality from COVID-19. In our study, the PLR did not emerge as a predictor of survival, suggesting reactive thrombocytosis as a suboptimal marker to identify severe COVID-19. This is perhaps unsurprising given that thrombocytopenia, rather than thrombocytosis, has been implicated in severe COVID-19.^26 27^

Our study shows that the systemic inflammatory response in patients with cancer diagnosed with COVID-19 is influenced by advanced age and comorbid burden. A number of studies have reproducibly shown these features to predict for adverse clinical course in SARS-CoV-2-infected patients irrespective of a diagnosis of cancer.^13 21–24^ Our data contribute to shed light on this relationship by highlighting that these categories at risk are more likely to mount an exaggerated innate response to SARS-CoV-2 as evidenced by significantly worse derangement biomarkers of systemic inflammation in these subgroups, tracing an important link between the extent of such proinflammatory response and outcome in these high-risk categories.

Because the panel of proinflammatory markers we studied relies on diverse and often non-redundant acute-phase reactants (hypoalbuminemia and neutrophilia) an important aim of our study was to establish the prognostic accuracy of each score in predicting patients’ OS.

We therefore tested the inflammatory indices in multivariable models including age, comorbid burden, and sex. We confirmed the combination of hypoalbuminemia and lymphopenia as computed by the OIS as a strong, independent predictor of survival, leading us to optimally select proinflammatory features that best scale with outcome in the context of COVID-19 and cancer. Both lymphopenia and hypoalbuminemia have previously been identified as independent prognostic factors in patients affected by COVID-19 without cancer.^27 28^ Our study shows that combination of these traits optimally scales with the prognosis of SARS-CoV-2-infected patients with cancer, an approach that was not considered in previous studies.

Taken together, our results qualify the OIS in SARS-CoV-2-infected patients with cancer as a readily available, inexpensive, and validated predictor of outcome in this patient population characterized by high risk of mortality following infection with SARS-CoV-2. The independence of and tumor-agnostic value of the OIS yields potential promise as a source of therapeutic targets to modulate innate immunity and prevent end-organ damage and COVID-19-related mortality in patients with cancer.

Our study acknowledges a number of limitations. The retrospective study design is a limitation shared by a number of studies published in this area^29–31^ and contributes to heterogeneity in treatment decisions, including escalation to intensive/high-dependence care and missing outcome data. Furthermore, non-cancer controls would have helped us understand whether the impact of proinflammatory scores is similar in patients who do not have cancer. Additionally, our study included only patients with confirmed SARS-CoV-2 infection determined by RT-PCR, leaving us no information on immune dysfunction in asymptomatic carriers. Finally, the 270-day gap between pre-COVID-19 and at COVID-19 diagnosis inflammatory index calculations for patients with pre-COVID-19, at, and post-COVID-19 diagnosis information available is a study limitation as is the smaller size of this 65-patient cohort.

Despite the acknowledged limitations, our study is the largest to attempt a robust and standardized evaluation of novel biomarkers of outcome in SARS-CoV-2-infected patients with cancer. The geographical diversity of our patient population and the choice of external validation limit the risk of overfitting of our survival estimates and facilitate a broader generalizability of our results.

In conclusion, we have demonstrated that the systemic inflammatory response can be used not only to facilitate an individualized risk assessment of patients’ mortality but also to better understand the disease biology of SARS-CoV-2 infection. Our work ties a dysfunctional proinflammatory response to poorer outcomes from COVID-19 in cancer and validates the combination of lymphopenia and hypoalbuminemia as measured by the OIS as a tumor-agnostic predictor of mortality following adjustment for key clinicopathological features, including comorbid burden and age. In the clinic, patients presenting with an OIS below 40 at COVID-19 diagnosis should be considered at higher risk of adverse outcome by treating physicians. Future study of the OIS in patients without cancer is warranted to validate its utility as a predictor of outcome in the general population. Our findings provide a clinical rationale for therapeutic targeting of the inflammatory response in SARS-CoV-2-infected patients with cancer.

## Data Availability

Data are available upon reasonable request.
