# Correction: *Systemic pro-inflammatory response identifies patients with cancer with adverse outcomes from SARS-CoV-2 infection: the OnCovid Inflammatory Score*

**DOI:** 10.1136/jitc-2020-002277corr1

**Published:** 2021-06-16

Dettorre GM, Dolly S, Loizidou A, On behalf of OnCovid study group *et al*. Systemic pro-inflammatory response identifies patients with cancer with adverse outcomes from SARS-CoV-2 infection: the OnCovid Inflammatory Score. *J Immunother Cancer* 2021;**9:**e002277. doi: 10.1136/jitc-2020-002277

This paper has been updated to amend author affiliations.

