# Supplementary material for: Systemic pro-inflammatory response identifies patients with cancer with adverse outcomes from SARS-CoV-2 infection: the OnCovid Inflammatory Score
Source: J Immunother Cancer. 2021 Mar 22;9(3):e002277. doi: 10.1136/jitc-2020-002277 (PMC7985977; doi:10.1136/jitc-2020-002277)
Supplement: Supplementary data [file jitc-2020-002277supp001.pdf]

Supplementary Table 1. Countries of origin and academic centers.

Academic Center	Number of Patients
**United Kingdom**	**539**
Barts Health NHS Trust (London)	124
Chelsea and Westminster Hospital NHS Foundation Trust (London)	114
Guy's and St Thomas' NHS Foundation Trust (London)	156
Imperial College Healthcare NHS Trust (London)	28
University College London Hospitals NHS Foundation Trust (London)	98
Velindre Cancer Center (Cardiff)	19
**Spain**	**380**
Catalan Institue of Oncology (Girona)	48
Hospital Clínic de Barcelona (Barcelona)	53
ICO L'Hospitalet de Llobregat (Barcelona)	86
Institut Català d'Oncologia Badalona (Barcelona)	50
Vall d'Hebron University Hospital (Barcelona)	143
**Italy**	**374**
Azienda Ospedaliera Nazionale SS. Antonio e Biagio e Cesare Arrigo (Alessandria)	21
Azienda Ospedaliera Spedali Civili di Brescia (Brescia)	26
Fondazione Poliambulanza Istituto Ospedaliero (Brescia)	10
Humanitas Cancer Center (Milan)	76
Istituto Nazionale Tumori (Milan)	6
Ospedale di Cremona (Cremona)	26
Ospedale Maggiore della Carità (Novara)	76
Ospedale Papa Giovanni XXIII (Bergamo)	107
Policlinico San Matteo (Pavia)	20
Università Campus Bio-Medico (Rome)	6
**Belgium**	**19**
Institut Jules Bordet (Brussels)	19
**Germany**	**6**
Medical Center of the University of Munich (Munich)	6

NHS: National Health Service; ICO: Institut Catala d'Oncologia
