# Supplementary material for: Systemic pro-inflammatory response identifies patients with cancer with adverse outcomes from SARS-CoV-2 infection: the OnCovid Inflammatory Score
Source: J Immunother Cancer. 2021 Mar 22;9(3):e002277. doi: 10.1136/jitc-2020-002277 (PMC7985977; doi:10.1136/jitc-2020-002277)
Supplement: Supplementary data [file jitc-2020-002277supp002.pdf]

Supplementary Table 2. Calculation of inflammation-based markers utilized for survival analyses. Formulas for calculation of inflammatory markers are described with cutoffs for placement into good, intermediate, and poor risk groups (0, 1, and 2 respectively).

Inflammation-based Marker	Score
**Neutrophil-lymphocyte ratio (NLR)**	
Total neutrophil count/total lymphocyte count; cutoff values utilized: 6:1 and ≥6:1	
6:1	0
≥6:1	1
**Platelet-lymphocyte ratio (PLR)**	
Total platelet count/total lymphocyte count; cutoff values utilized: 270:1 and ≥270:1	
270:1	0
≥270:1	1
**OnCovid Inflammatory Score (OIS)**	
Albumin (g/L) + 5 × lymphocytes (10^9/L); cutoff values utilized: ≤40 and 40	
40	0
≤40	1
**Modified Glasgow prognostic score (mGPS)**	
CRP ≤10 mg/L	0
CRP 10mg/L and albumin ≥ 35 g/L	1
CRP 10mg/L and albumin 35 g/L	2
**Prognostic index (PI)**	
CRP ≤ 10mg/L and WCC ≤ 11 (10^9/L)	0
CRP ≤ 10mg/L and WCC 11 (10^9/L)	1
CRP 10mg/L and WCC ≤ 11 (10^9/L)	1
CRP 10mg/L and WCC 11 (10^9/L)	2

NLR: Neutrophil-lymphocyte ratio; PLR: Platelet-lymphocyte ratio; OIS: OnCovid Inflammatory Score; mGPS: Modified Glasgow prognostic score; CRP: C-reactive protein; PI: Prognostic index; WCC: White cell count
