# Supplementary material for: Systemic pro-inflammatory response identifies patients with cancer with adverse outcomes from SARS-CoV-2 infection: the OnCovid Inflammatory Score
Source: J Immunother Cancer. 2021 Mar 22;9(3):e002277. doi: 10.1136/jitc-2020-002277 (PMC7985977; doi:10.1136/jitc-2020-002277)
Supplement: Supplementary data [file jitc-2020-002277supp003.pdf]

Supplementary Table 3. Training and validation set matching for key characteristics. *P* values for differences in categorical distributions of known risk factors for severe Covid-19 for training (*n*=529) and validation (*n*=542) sets determined via Pearson's chi-square test.

Characteristic	X^2; *P* value Between Training and Validation Sets
Proportion below 65 versus at or above 65 years of age	0.697 (1 df); 0.404
Number of comorbidities (0, 1, 2, 3+)	2.006 (3 df); 0.571
Number of Covid-19 complications (0, 1, 2, 3+)	0.940 (3 df); 0.816
Proportion with active malignancy versus in remission	3.445 (1 df); 0.063

Covid-19: Coronavirus disease 2019; df: Degrees of freedom
