# Supplementary material for: Systemic pro-inflammatory response identifies patients with cancer with adverse outcomes from SARS-CoV-2 infection: the OnCovid Inflammatory Score
Source: J Immunother Cancer. 2021 Mar 22;9(3):e002277. doi: 10.1136/jitc-2020-002277 (PMC7985977; doi:10.1136/jitc-2020-002277)
Supplement: Supplementary data [file jitc-2020-002277supp004.pdf]

Supplementary Table 4. Patient demographics of whole dataset.

**Characteristic**	**Dataset** (Total, *n* =1,071)
Age (years), mean ± SD	68.2 ± 13.4
Age ≥65 years, no. (%)	645 (60.2)
Sex, no. (%)	
Male	625 (58.3)
Female	443 (41.4)
Information unavailable	3 (0.3)
Smoking history, no. (%)	
Never smoker	412 (38.5)
Current/former smoker	462 (43.1)
Unknown	197 (18.4)
Cancer type, no. (%)	
Head & neck	34 (3.2)
Lung & thoracic	154 (14.4)
Gastroesophageal	52 (4.9)
Hepatobiliary	55 (5.1)
Duodenal & lower GI tract	132 (12.3)
Breast	177 (16.5)
Gynecological	57 (5.3)
Genitourinary	216 (20.2)
Skin	43 (4.0)
Lymphoma	87 (8.1)
Other	64 (6.0)
Tumor stage, no. (%)	
Localized	360 (33.6)
Locoregional	159 (14.8)
Metastatic	420 (39.2)
Information unavailable	132 (12.4)
Tumor status at Covid-19 diagnosis, no. (%)	
Active malignancy	687 (64.1)
Remission	332 (31.0)
Information unavailable	52 (4.9)
Ongoing anticancer therapy at Covid-19 diagnosis, no. (%)	516 (48.2)
Prior radical therapies, no. (%)	591 (55.2)
Surgery	510 (47.6)
Adjuvant/neoadjuvant chemotherapy	319 (29.8)
Prior palliative systemic therapy, no. (%)	277 (25.9)
Chemotherapy	184 (17.2)
Immunotherapy	46 (4.3)
Endocrine therapy	53 (4.9)
Targeted therapy	45 (4.2)
Prior curative systemic therapy, no. (%)	62 (5.8)
Prior radiotherapy, no. (%)	319 (29.8)
Prior lines of palliative therapy, no. (%)	
1	135 (12.6)
2	59 (5.5)
≥3	61 (5.7)
Comorbidities, no. (%)	844 (78.8)
Hypertension	496 (46.3)
Diabetes	238 (22.2)
Cardiovascular disease	257 (24.0)
Chronic pulmonary disease	160 (14.9)
Chronic kidney disease	125 (11.7)
Cerebrovascular disease	78 (7.3)
Dementia	63 (5.9)
Peripheral vascular disease	40 (3.7)
Liver impairment	21 (2.0)
Immunosuppression	45 (4.2)

Steroid therapy in progress	50 (4.7)
Other	308 (28.8)
Number of comorbidities, no. (%)	
0	227 (21.2)
1	298 (27.8)
2	247 (23.1)
≥3	299 (27.9)
Covid-19 symptoms at diagnosis, no. (%)	1011 (94.4)
Fever	660 (61.6)
Cough	542 (50.6)
Dyspnea	438 (40.9)
Fatigue	257 (24.0)
Myalgia	117 (10.9)
Diarrhea	126 (11.8)
Coryzal symptoms	49 (4.6)
Nausea or vomiting	80 (7.5)
Sore throat	27 (2.5)
Headache	42 (3.9)
Dysgeusia	35 (3.3)
Anosmia	29 (2.7)
Other (i.e. confusion, delirium, etc.)	243 (22.7)
Number of symptoms at diagnosis, no. (%)	
0	60 (5.6)
1	229 (21.4)
2	300 (28.0)
≥3	482 (45.0)
Hospitalization rate, no. (%)	957 (89.4)
Admission to intensive or sub-intensive care unit, no. (%)	124/957 (13.0)
Covid-19-specific drug treatments, no. (%)	708 (66.1)
Antibiotics	595 (55.6)
Hydroxychloroquine or chloroquine	392 (36.6)
Lopinavir/ritonavir	172 (16.1)
Systemic corticosteroids	91 (8.5)
Remdesivir	12 (1.1)
Tocilizumab	46 (4.3)
Others	128 (12.0)
Covid-19-specific oxygen interventions, no. (%)	632 (59.0)
Oxygen therapy	627 (58.5)
Mechanical ventilation	115 (10.7)
Covid-19 complications, no. (%)	754 (70.4)
Acute respiratory failure	627 (58.5)
ARDS	131 (12.2)
Acute kidney injury	86 (8.0)
Secondary infection	86 (8.0)
Sepsis	52 (4.9)
Septic shock	45 (4.2)
Acute cardiac injury	24 (2.2)
Acute liver injury	10 (0.9)
Others (i.e. DIC, etc.)	61 (5.7)
Number of complications, no. (%)	
0	317 (29.6)
1	394 (36.8)
2	194 (18.1)
≥3	89 (8.3)
Information unavailable	77 (7.2)

SD: Standard deviation; GI: Gastrointestinal; Covid-19: Coronavirus disease 2019; ARDS: Acute respiratory distress syndrome; DIC: Disseminated intravascular coagulation
