# Supplementary material for: Systemic pro-inflammatory response identifies patients with cancer with adverse outcomes from SARS-CoV-2 infection: the OnCovid Inflammatory Score
Source: J Immunother Cancer. 2021 Mar 22;9(3):e002277. doi: 10.1136/jitc-2020-002277 (PMC7985977; doi:10.1136/jitc-2020-002277)

Supplementary Figure 1. Inflammatory marker values versus status of malignancy. Median inflammatory index values or distributions at Covid-19 diagnosis are displayed against the status of malignancy. Values and distributions shown for the NLR (*n*=667 active, *n*=320 remission; *P*=0.50), PLR (*n*=643 active, *n*=317 remission; *P*=0.32), OIS (*n*=418 active, *n*=199 remission; *P*0.05), mGPS (*n*=414 active, *n*=228 remission; *P*0.05), and PI (*n*=528 active, *n*=259 remission; *P*=0.40). *=*P*0.05. Error bars represent 95% confidence interval from the median.

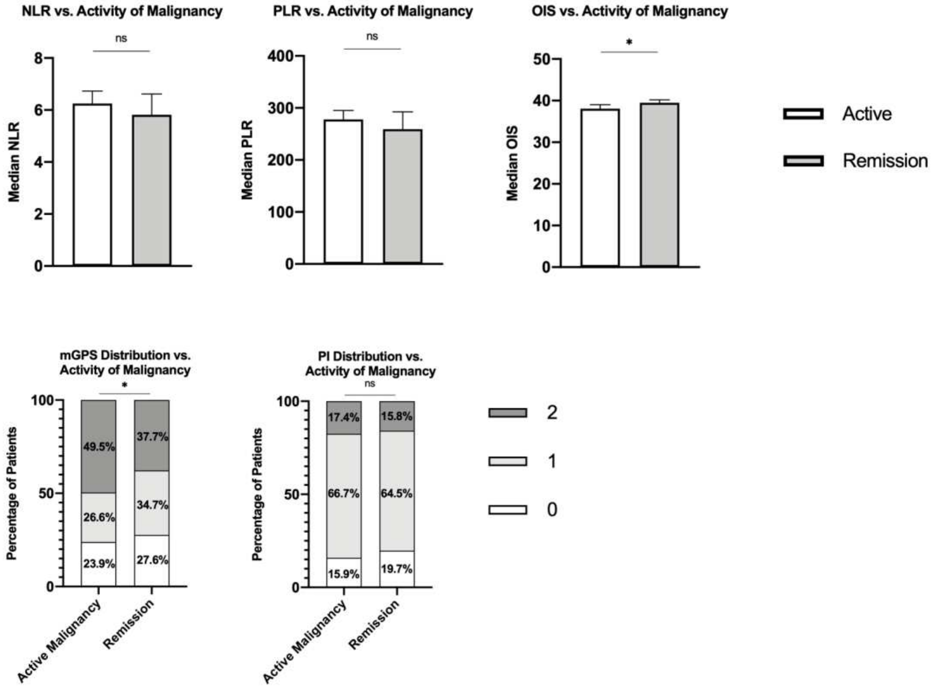

Supplement: Supplementary data [file jitc-2020-002277supp009.pdf]
