# Supplementary material for: Systemic pro-inflammatory response identifies patients with cancer with adverse outcomes from SARS-CoV-2 infection: the OnCovid Inflammatory Score
Source: J Immunother Cancer. 2021 Mar 22;9(3):e002277. doi: 10.1136/jitc-2020-002277 (PMC7985977; doi:10.1136/jitc-2020-002277)
Supplement: Supplementary data [file jitc-2020-002277supp005.pdf]

Supplementary Table 5. Inflammatory marker values across cancer types. Unadjusted mortality, median inflammatory marker values with interquartile ranges, and categorically distributed inflammatory marker values shown for primary tumor site categories of the whole dataset (*n*=1,071).

Tumor Site	Unadjusted Mortality	NLR (median, IQR)	PLR (median, IQR)	OIS (median, IQR)	mGPS (0/1/2, %)	PI (0/1/2, %)
**Head & Neck**	38.2%	7.1 (10.8)	390.4 (245.0)	39.8 (9.2)	5/6/11 (22.7%/27.3%/50.0%)	5/21/3 (17.2%/72.4%/10.3%)
**Lung & Thoracic**	47.4%	7.8 (9.4)	296.3 (249.3)	37.8 (9.7)	21/23/38 (25.6%/28.0%/46.3%)	13/86/26 (10.4%/68.8%/20.8%)
**Gastroesophageal**	32.7%	8.2 (12.5)	238.0 (415.4)	38.4 (10.6)	8/5/18 (25.8%/16.1%/58.1%)	7/19/10 (19.4%/52.8%/27.8%)
**Hepatobiliary**	47.3%	6.4 (8.3)	212.5 (265.6)	33.8 (10.8)	13/5/18 (36.1%/13.9%/50.0%)	12/25/10 (25.5%/53.2%/21.3%)
**Duodenal & Lower GI Tract**	38.6%	6.3 (9.2)	309.7 (221.3)	36.5 (10.5)	18/16/42 (23.7%/21.1%/55.3%)	16/62/13 (17.6%/68.1%/14.3%)
**Breast**	20.9%	4.3 (5.9)	221.7 (202.3)	39.8 (8.4)	36/37/33 (34.0%/34.9%/31.1%)	33/89/14 (24.3%/65.4%/10.3%)
**Gynecological**	36.8%	4.9 (5.9)	282.6 (273.6)	36.7 (10.3)	5/9/24 (13.2%/23.7%/63.2%)	5/35/8 (10.4%/72.9%/16.7%)
**Genitourinary**	34.3%	6.6 (10.7)	271.9 (266.0)	38.0 (8.7)	26/51/78 (16.8%/32.9%/50.3%)	18/123/29 (10.6%/72.4%/17.1%)
**Skin**	39.5%	6.1 (9.0)	217.5 (276.6)	42.5 (15.0)	3/20/11 (8.8%/58.8%/32.4%)	1/26/8 (2.9%/74.3%/22.9%)
**Lymphoma**	33.3%	4.8 (5.8)	214.8 (335.5)	40.0 (11.1)	22/14/21 (38.6%/24.6%/36.8%)	20/32/8 (33.3%/53.3%/13.3%)
**Other**	26.6%	5.6 (9.4)	255.5 (284.9)	39.1 (10.6)	12/10/13 (34.3%/28.6%/37.1%)	12/28/11 (23.5%/54.9%/21.6%)

NLR: Neutrophil-lymphocyte ratio; PLR: Platelet-lymphocyte ratio; OIS: OnCovid Inflammatory Score; mGPS: Modified Glasgow prognostic score; PI: Prognostic index; IQR: Interquartile range; GI: Gastrointestinal
