# Supplementary material for: Systemic pro-inflammatory response identifies patients with cancer with adverse outcomes from SARS-CoV-2 infection: the OnCovid Inflammatory Score
Source: J Immunother Cancer. 2021 Mar 22;9(3):e002277. doi: 10.1136/jitc-2020-002277 (PMC7985977; doi:10.1136/jitc-2020-002277)
Supplement: Supplementary data [file jitc-2020-002277supp006.pdf]

Supplementary Table 6. Associations between inflammatory markers and predictors of mortality or disease severity. Inflammatory marker risk groups tested for association with known predictors of mortality from or development of severe Covid-19 for the whole dataset (*n*=1,071). *P* values shown for strength of association determined via Pearson's chi-square test.

Inflammatory Marker	# of Comorbidities (0/1/2+, %)	*P* Value	# of Complications (0/1/2+, %)	*P* Value	Anti-cancer Therapy at Diagnosis (N/Y %)	*P* Value	Sex (F/M, %)	*P* value	Age (65/≥65, %)	*P* value
**NLR**										
NLR6	133/147/230 (26.1%/28.8%/45.1%)	***.0001***	191/176/111 (40.0%/36.8%23.2%)	***.0001***	216/274 (44.1%/55.9%)	***.001***	243/264 (47.9%/52.1%)	***.0001***	235/274 (46.2%/53.8%)	***.0001***
NLR≥6	86/141/299 (16.3%/26.8%/56.8%)		117/205/165 (24.0%/42.1%/33.9%)		282/228 (55.3%/44.7%)		187/339 (35.6%/64.4%)		172/350 (33.0%/67.0%)	
**PLR**										
PLR270	109/150/230 (22.3%/30.7%/47.0%)	0.064	163/177/115 (35.8%/38.9%/25.3%)	***0.020***	236/246 (49.0%/51.0%)	0.747	214/273 (43.9%/56.1%)	0.212	202/282 (41.7%, 58.3%)	0.069
PLR≥270	99/125/267 (20.2%/25.5%/54.4%)		134/183/152 (28.6%/39.0%/32.4%)		243/243 (50.0%/50.0%)		196/294 (40.0%/60.0%)		177/314 (36.0%/64.0%)	
**OIS**										
OIS40	69/76/124 (25.7%/28.3%/46.1%)	***0.010***	104/102/46 (41.3%/40.5%/18.3%)	***.0001***	127/129 (49.6%/50.4%)	0.124	115/154 (42.8%/57.2%)	0.152	115/154 (42.8%/57.2%)	***0.049***
OIS≤40	71/91/222 (18.5%/23.7%/57.8%)		88/130/134 (25.0%/36.9%/38.1%)		205/162 (55.9%/44.1%)		142/240 (37.2%/62.8%)		135/249 (35.2%/64.8%)	
**mGPS**										
0	50/53/66 (29.6%/31.4%/39.1%)		73/62/26 (45.3%/38.5%/16.1%)		75/91 (45.2%/54.8%)		82/87 (48.5%/51.5%)		85/84 (50.3%/49.7%)	
1	52/49/95 (26.5%/25.0%/48.5%)	***.001***	58/85/39 (31.9%/46.7%/21.4%)	***.0001***	99/91 (52.1%/47.9%)	**0.005**	80/116 (40.8%/59.2%)	**0.036**	79/117 (40.3%/59.7%)	**0.004**
2	52/73/182 (16.9%/23.8%/59.3%)		64/98/118 (22.9%/35.0%/42.1%)		178/116 (60.5%/39.5%)		111/194 (36.4%/63.6%)		106/201 (34.5%/65.5%)	
**PI**										
0	45/43/54 (31.7%/30.3%/38.0%)		62/54/20 (45.6%/39.7%/14.7%)		59/80 (42.4%/57.6%)		70/72 (49.3%/50.7%)		73/69 (51.4%/48.6%)	
1	102/149/295 (18.7%/27.3%/54.0%)	**0.002**	126/209/170 (25.0%/41.4%/33.7%)	***.0001***	245/282 (46.5%/53.5%)	***.0001***	218/326 (40.1%/59.9%)	**0.139**	209/332 (38.6%/61.4%)	**0.001**
2	24/43/73 (17.1%/30.7%/52.1%)		26/54/51 (19.8%/41.2%/38.9%)		94/40 (70.1%/29.9%)		58/82 (41.4%/58.6%)		43/97 (30.7%/69.3%)	

N: No; Y: Yes; F: Female; M: Male; NLR: Neutrophil-lymphocyte ratio; PLR: Platelet-lymphocyte ratio; OIS: OnCovid Inflammatory Score; mGPS: Modified Glasgow prognostic score; PI: Prognostic index
