# Supplementary material for: Systemic pro-inflammatory response identifies patients with cancer with adverse outcomes from SARS-CoV-2 infection: the OnCovid Inflammatory Score
Source: J Immunother Cancer. 2021 Mar 22;9(3):e002277. doi: 10.1136/jitc-2020-002277 (PMC7985977; doi:10.1136/jitc-2020-002277)

Supplementary Figure 2. Relationship between inflammatory markers and clinical variables. Median values for clinical variables are shown for patients in good and poor risk groups for each inflammatory index. *=*P*0.05; **=*P*0.01; ***=*P*0.001; ****=*P*0.0001. Error bars represent 95% confidence interval from the median.

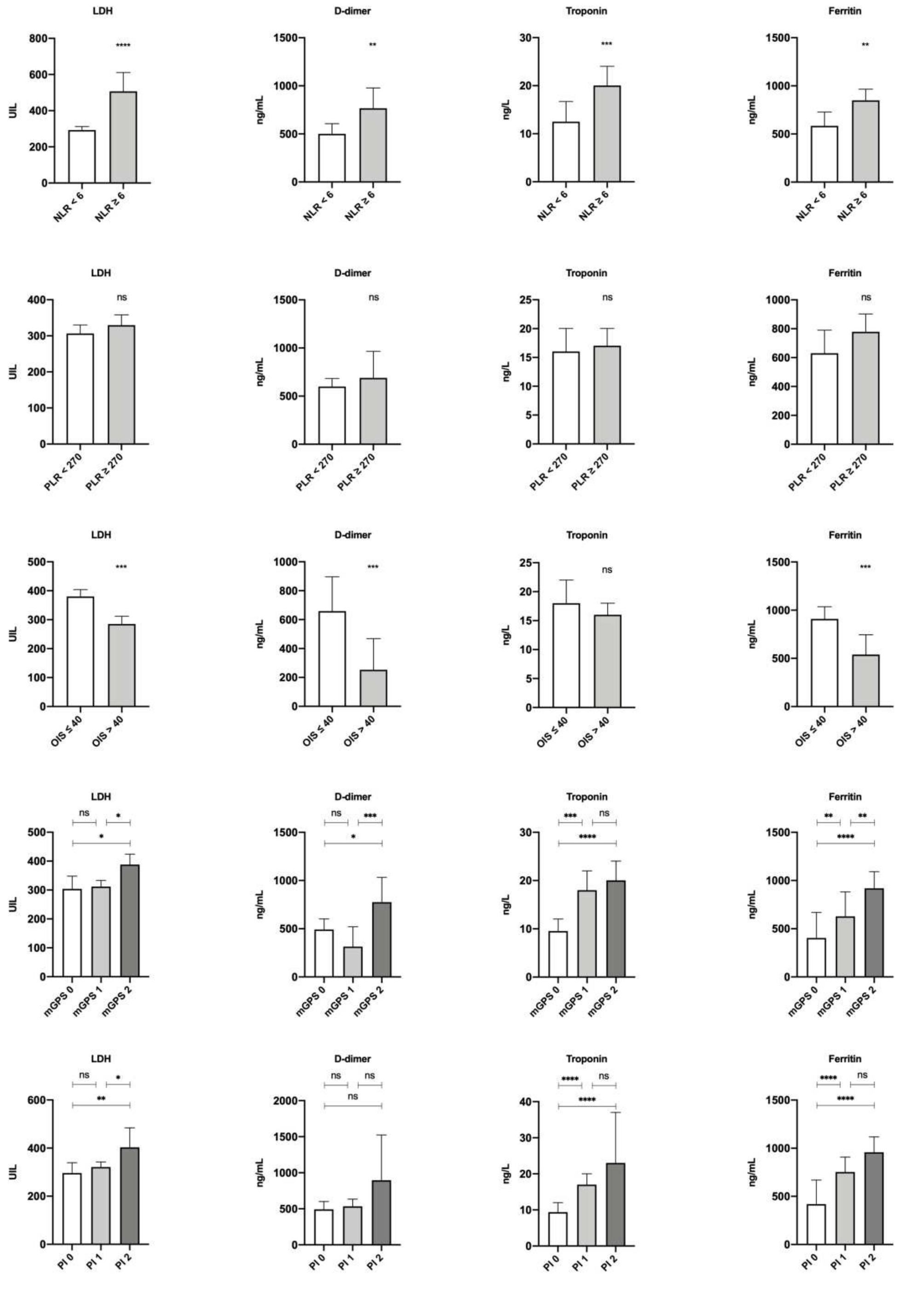

Supplement: Supplementary data [file jitc-2020-002277supp010.pdf]
