# Supplementary material for: Systemic pro-inflammatory response identifies patients with cancer with adverse outcomes from SARS-CoV-2 infection: the OnCovid Inflammatory Score
Source: J Immunother Cancer. 2021 Mar 22;9(3):e002277. doi: 10.1136/jitc-2020-002277 (PMC7985977; doi:10.1136/jitc-2020-002277)
Supplement: Supplementary data [file jitc-2020-002277supp007.pdf]

Supplementary Table 7. Inflammatory markers at Covid-19 diagnosis and median overall survival. Median overall survival, 95% confidence intervals, and *P* values determined via Kaplan-Meier estimates with log-rank methodology for training (*n*=529) and validation (*n*=542) sets. **=*P*0.01; ***=*P*0.001; ****=*P*0.0001.

Inflammatory Marker	Median OS; 95% CI (Training Set)	Univariable *P* Value	Median OS; 95% CI (Validation Set)	Univariable *P* Value
**NLR**				
NLR6	Not reached	0.001***	Not reached	0.0001****
NLR≥6	30 days; 1-63 days		41 days; 15-67 days	
**OIS**				
OIS40	Not reached	0.0001****	Not reached	0.0001****
OIS≤40	23 days; 10-35 days		40 days; 8-72 days	
**mGPS**				
0	Not reached	0.0001****	Not reached	0.001***
1	Not reached		Not reached	
2	20 days; 8-32 days		41 days; 8-74 days	
**PI**				
0	Not reached	0.0001****	Not reached	0.01**
1	81 days; 24-138 days		271 days; incalculable	
2	23 days; 1-56 days		22 days; 0-61 days	

OS: Overall survival; CI: Confidence interval; NLR: Neutrophil-lymphocyte ratio; OIS: OnCovid Inflammatory Score; mGPS: Modified Glasgow prognostic score; PI: Prognostic index
