# Supplementary material for: Systemic pro-inflammatory response identifies patients with cancer with adverse outcomes from SARS-CoV-2 infection: the OnCovid Inflammatory Score
Source: J Immunother Cancer. 2021 Mar 22;9(3):e002277. doi: 10.1136/jitc-2020-002277 (PMC7985977; doi:10.1136/jitc-2020-002277)
Supplement: Supplementary data [file jitc-2020-002277supp008.pdf]

Supplementary Table 8. C-index statistics for inflammatory markers. Harrell's C-index results are shown along with optimism-adjusted C-indices and 95% confidence intervals for training (*n*=529) and validation (*n*=542) sets.

	**Training**	***n*=529**				**Validation**	***n*=542**			
**Prognostic Factor**	**C-index**	**95% CI**	**Optimism**	**Adjusted C-index**	**95% CI**	**C-index**	**95% CI**	**Optimism**	**Adjusted C-index**	**95% CI**
**NLR**	0.567	.534 - .603	0.0029	0.564	.531 - .600	0.587	.556 - .624	0.0018	0.585	.554 - .622
**PLR**	0.505	.492 - .553	0.0120	0.493	.480 - .541	0.551	.514 - .592	-0.0006	0.552	.514 - .593
**OIS**	0.606	.557 - .648	0.0022	0.603	.555 - .646	0.611	.564 - .668	0.0002	0.611	.564 - .668
**mGPS**	0.637	.597 - .682	0.0027	0.634	.595 - .680	0.600	.553 - .655	0.0043	0.596	.548 - .651
**PI**	0.581	.551 - .624	0.0035	0.577	.548 - .620	0.574	.532 - .623	0.0004	0.573	.532 - .622
**Multivariable model**	0.670	.642 - .753	0.0286	0.642	.613 - .724	0.690	.661 - .790	0.0317	0.659	.629 - .758

CI: Confidence interval; NLR: Neutrophil-lymphocyte ratio; PLR: Platelet-lymphocyte ratio; OIS: OnCovid Inflammatory Score; mGPS: Modified Glasgow prognostic score; PI: Prognostic index
